# Translating Osteoarthritis Genetic Risk Into Biomarkers: Opportunities, Pitfalls, and Implementation Considerations

**DOI:** 10.1155/humu/3061956

**Published:** 2026-06-17

**Authors:** Tao Meng, Lina Ma, Xiaoqing Zhang, Jinchang Han, Fuyuan Li, Weiyong Wu, Shilong Liu, Aifeng Liu

**Affiliations:** ^1^ Department of Orthopedics, First Teaching Hospital of Tianjin University of Traditional Chinese Medicine, Tianjin, China, tjtcm.cn; ^2^ National Clinical Research Center for Chinese Medicine, Tianjin, China; ^3^ Department of Pharmacy, First Teaching Hospital of Tianjin University of Traditional Chinese Medicine, Tianjin, China, tjtcm.cn

**Keywords:** biomarker translation, clinical implementation, eQTL/pQTL, fine-mapping, genetic risk, osteoarthritis, polygenic risk score, single-cell multiomics, spatial transcriptomics, variant interpretation

## Abstract

Osteoarthritis (OA) is a heterogeneous joint disease in which patients differ widely in onset, progression, pain burden, and inflammatory activity, yet clinical tools for early risk stratification and mechanism‐informed subtyping remain limited. Human genetic studies have identified many OA‐associated loci, but translation into deployable biomarkers has been slow because most signals are polygenic, largely noncoding, and dependent on tissue and cell state. Despite rapid progress in OA genomics, a major remaining challenge is how to systematically convert genetic discoveries into clinically actionable biomarkers that can support risk prediction, biological stratification, and therapeutic development. This review discusses how OA genetic risk can be converted into practical biomarker strategies by combining statistical variant interpretation with joint‐resolved biology. We summarize approaches that prioritize likely effector genes and regulatory modes using fine‐mapping and molecular QTL evidence, and then place these signals into the correct anatomical and cellular contexts using single‐cell and spatial atlases of cartilage, synovium, and subchondral bone. We highlight three classes of outputs that are most likely to be clinically useful: polygenic risk‐informed stratification for early monitoring and trial enrichment; compact molecular panels reflecting genetically supported programs in accessible biospecimens; and imaging–omics models that connect structural phenotypes to mechanism‐linked biology. We also review common reasons biomarker pipelines fail in OA, including uncertain variant‐to‐gene assignment, limited portability across populations, tissue accessibility and stage bias, and technical variation across omics and imaging platforms. Finally, we outline what is needed for responsible deployment—standardized assays, clinically meaningful evaluation, external replication in diverse cohorts, and clear governance for privacy, consent, and model accountability.

## 1. Introduction

Osteoarthritis (OA) is the most common chronic joint disease and a major cause of pain, disability, and loss of mobility [1]. Despite its burden, management remains largely reactive: symptoms are treated, and structural damage is often addressed late [2]. One reason is that OA is not a single biological entity [3]. Patients with similar radiographic changes can follow very different courses, with wide variation in progression rate, pain severity, inflammatory features, and functional decline [4, 5]. These differences point to multiple, partially overlapping disease processes that play out across cartilage, synovium, subchondral bone, and other joint compartments [6]. This biological complexity represents a major barrier to precision medicine in OA, as current clinical tools rarely capture the underlying mechanistic diversity that drives disease heterogeneity.

Human genetics offers a promising starting point for improving this situation [7]. Genetic signals are present before clinical onset, are less vulnerable to reverse causation, and can support more credible causal hypotheses than many observational biomarker studies [8]. In principle, genetics should help identify patients at higher liability earlier, and it should also highlight pathways and cell programs that define biologically meaningful subgroups [9]. In practice, however, translating OA loci into biomarkers has been difficult. Most associations have modest effects, are distributed across many loci, and lie outside coding regions [10]. As a result, the key questions are often unanswered: which genes and regulatory mechanisms are truly involved, in which joint tissues they matter, and what measurable readouts can reliably capture those processes [11]. More importantly, even when genetic mechanisms are identified, a systematic framework for converting these findings into clinically actionable biomarkers remains underdeveloped.

Recent advances make this translation problem more tractable than it was even a few years ago [9]. Fine‐mapping and molecular quantitative trait loci (QTL) resources can narrow candidate mechanisms and suggest likely effector genes, whereas single‐cell and spatial profiling can localize those mechanisms to specific cell types, states, and microanatomic niches within the joint [12, 13]. This matters for OA because relevant tissue is rarely sampled until late‐stage surgery, and bulk measurements can easily dilute compartment‐restricted programs. The combination of statistical genetics with joint‐resolved atlases therefore provides a practical route for moving from risk loci to biomarker candidates that are mechanistically interpretable and realistically measurable [14]. Importantly, these developments now allow genetic discoveries to be evaluated not only for biological insight but also for their potential translational value in biomarker development and patient stratification.

**Figure 1:**
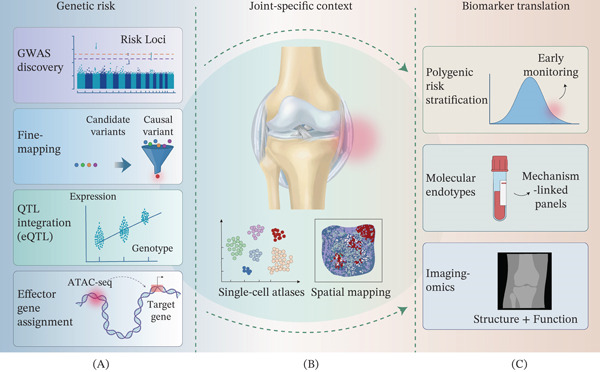
Conceptual workflow for translating osteoarthritis genetic risk into clinically deployable biomarkers. Genetic discovery and variant interpretation are linked to joint‐resolved single‐cell/spatial context to enable polygenic risk stratification, mechanism‐linked molecular endotypes, and imaging–omics signatures.

In this review, we focus on how to translate OA genetic risk into biomarkers that can be evaluated and deployed. Rather than providing a conventional overview of OA genetics, this review aims to frame recent advances within a genetics‐to‐biomarker translation perspective, emphasizing how genetic discoveries can inform practical biomarker strategies and clinical research design. We first summarize what OA genetic architecture implies for biomarker design. We then discuss approaches for variant interpretation and effector gene prioritization, and how single‐cell and spatial atlases can place genetically supported programs into the right tissue contexts. Finally, we consider how these insights can guide the construction of deployable outputs—polygenic risk‐informed stratification, compact molecular panels, and imaging–omics models—while addressing common pitfalls and the practical requirements for implementation. By integrating genetic evidence, tissue biology, and translational design principles, this review provide a conceptual roadmap for advancing precision biomarker development in OA (Figure 1).

## 2. Genetic Architecture of OA and What “Genetic Risk” Means for Biomarkers

### 2.1. Polygenic Susceptibility and Clinical Heterogeneity

OA has a measurable heritable component, but its genetic basis is largely complex rather than single‐gene [15]. In most individuals, liability reflects the cumulative contribution of many common variants with modest effects, alongside smaller contributions from less frequent variants and, in specific contexts, rare coding changes [16]. This architecture sets realistic expectations for translation: individual loci seldom provide enough separation to function as stand‐alone clinical tests [17]. Instead, genetic information in OA is more realistically viewed as providing probabilistic risk context rather than deterministic prediction, which has important implications for how biomarkers should be positioned clinically. It also intersects with the heterogeneity of OA itself [18]. Knee, hip, hand, and spine OA differ in pathobiology and clinical course, and genetic associations may align more strongly with particular outcomes—such as incident disease, osteophyte burden, cartilage‐related traits, inflammatory features, or pain‐related phenotypes—depending on how cases and endpoints are defined [19]. For example, genetic variants associated with structural OA may not necessarily predict pain severity, whereas variants linked to inflammatory pathways may be more relevant to symptomatic progression rather than radiographic change. Such distinctions illustrate why genetically informed biomarkers may perform differently depending on the clinical endpoint being evaluated. For biomarker development, clarifying the target phenotype is therefore not a formality; it determines whether a candidate marker is being asked to predict long‐term susceptibility, near‐term progression, symptom burden, or a mechanism‐linked subtype [20]. From a translational standpoint, this also means that genetic heterogeneity may necessitate different biomarker strategies—for example, susceptibility‐oriented markers for early identification versus activity‐related markers for progression monitoring.

### 2.2. Predominantly Regulatory Signals and Tissue Dependence

A large fraction of OA‐associated signals map to noncoding regions, consistent with regulatory mechanisms rather than protein‐altering changes [21]. In biomarker terms, this is consequential because variants acting through gene regulation often produce effects that depend on the relevant tissue compartment and cellular state [22]. A genetic association can be real while its molecular consequence is difficult to detect in an accessible sample, or visible only in a narrow joint niche or disease stage [23]. This helps explain why locus discovery has not automatically produced deployable biomarkers in OA. Translation typically requires identifying downstream readouts that reflect the regulatory program implicated by genetics, while being measurable with acceptable stability and reproducibility [16]. It also requires stating, explicitly, which compartment is most relevant—cartilage, synovium, subchondral bone, or mixed tissue interactions—because the same locus can plausibly play different roles across these contexts [22]. For example, regulatory variants influencing synovial inflammatory pathways may be more likely to yield detectable protein biomarkers in synovial fluid or circulation, whereas variants affecting cartilage homeostasis may be better reflected by imaging or cartilage turnover markers. Recognizing this tissue dependence is therefore essential for selecting clinically measurable biomarker surrogates.

### 2.3. Practical Roles of Genetic Information in Biomarker Development

In OA, genetic information tends to support biomarker development indirectly, rather than replacing molecular or imaging measures [17]. One role is enrichment and early stratification: polygenic risk scores (PRS) can identify individuals with higher baseline liability who may benefit from earlier monitoring or inclusion in prevention‐oriented studies, even if these scores are not sufficient for diagnosis on their own [17]. In practical terms, this may include identifying individuals at elevated genetic risk for closer longitudinal monitoring or for enrollment into prevention‐focused clinical trials rather than immediate therapeutic decision‐making. A second role is prioritization of biology: genetic evidence can narrow the search space by highlighting candidate effector genes and pathways that are more likely to be causal, which is valuable when multiomics analyses otherwise yield long lists that are difficult to reproduce [21]. A third role is improving interpretability and robustness when integrating modalities [24]. When candidate features are aligned with genetically supported, joint‐relevant programs, multimodal signatures are less likely to be driven by cohort‐specific correlates [24]. This is particularly relevant in OA where biomarker studies often face reproducibility challenges due to disease heterogeneity and technical variability. Finally, population transferability must be treated as a central requirement [25]. Differences in allele frequencies and linkage disequilibrium can reduce the portability of genetic scores, and this directly affects the credibility of any genetics‐informed biomarker [26]. For this reason, calibration, external replication, and evaluation across diverse populations should be considered part of biomarker design, not an afterthought [27].

## 3. From Loci to Effector Genes: Variant Interpretation and Prioritization

### 3.1. Fine‐Mapping as a Foundation for Downstream Interpretation

Most OA associations implicate genomic regions shaped by linkage disequilibrium rather than a single causal variant [28]. Fine‐mapping addresses this by refining each locus into a smaller set of plausible variants and, importantly, quantifying the remaining uncertainty (e.g., through posterior probabilities) [28]. For translational interpretation, the practical value of fine‐mapping lies in defining which variants warrant functional follow‐up and in reducing the risk of attributing biological meaning to a lead SNP that may only represent a proxy signal. In OA, where effect sizes are typically modest and linkage patterns can differ across ancestries, uncertainty‐aware fine‐mapping is especially important for avoiding false precision and ensuring reproducibility across cohorts with different genetic backgrounds.

### 3.2. Molecular QTL Evidence and Colocalization to Nominate Effector Genes

Because many OA signals are noncoding, connecting loci to effector genes is often the key bottleneck [29]. Molecular QTL resources—expression QTLs (eQTLs), splicing QTLs (sQTLs), and protein QTLs (pQTLs)—provide a direct route to infer which molecular traits are perturbed by candidate variants and in what direction [23, 30]. Colocalization analyses strengthen this inference by evaluating whether the OA association and the molecular association are likely driven by the same underlying genetic signal rather than by coincidental overlap within a linkage block [31]. From a biomarker development standpoint, QTL evidence can also redirect attention from the variant itself toward downstream molecular traits that are more directly measurable. At the same time, QTL interpretation should remain cautious: signals derived from nonjoint tissues or from bulk measurements may miss joint‐relevant, cell‐state–specific regulation, and tissue mismatch is a common reason variant‐to‐gene assignments fail to translate [12].

### 3.3. Functional Annotation in Joint‐Relevant Contexts

Functional annotation translates statistical candidates into mechanistic plausibility. For noncoding candidates, this typically prioritizes variants overlapping regulatory elements, variants predicted to alter transcription factor binding, and variants situated within active chromatin in relevant cell types [32]. In OA, however, regulatory relevance depends strongly on joint context, making annotations derived from cartilage, synovium, or subchondral bone more informative than those inferred from unrelated tissues. Three‐dimensional genome information and enhancer–promoter links can further refine gene assignments when multiple genes reside near a locus [33]. For coding candidates, interpretation relies more on predicted effects on protein function, evolutionary constraint, and concordance with curated variant knowledge. Across both classes, the aim is to specify a plausible biological mechanism that can be tested—where and when the effect is expected to occur, and what molecular consequence should be observed—rather than relying on generic regulatory annotations that do not substantially narrow functional hypotheses.

### 3.4. Prioritizing Signals That Are Both Credible and Assayable

Not every statistically supported locus yields a biomarker opportunity. A translation‐oriented prioritization step is therefore necessary, focusing on signals that show convergent support across fine‐mapping, molecular QTL integration, and functional context, while also offering a realistic path to measurement [9]. Some mechanisms naturally point to tractable readouts, such as protein abundance changes, stable isoform shifts, or pathway activity signatures that can be captured in accessible biospecimens or imaging features. Other signals may be biologically important yet difficult to measure or too compartment‐restricted to translate reliably. In OA, prioritization should also consider disease stage and tissue accessibility, because mechanisms apparent in late‐stage surgical tissues may not support early‐risk biomarkers, and circulating markers can be dominated by systemic variation [12]. Explicitly distinguishing biological relevance from practical measurability helps focus biomarker development on candidates that can ultimately be standardized and clinically evaluated.

## 4. Mapping Genetic Risk to Joint Tissues and Cell States With Single‐Cell and Spatial Atlases

### 4.1. The Joint Is Not a Single Tissue

A recurring obstacle in OA translation is the tendency to treat the disease as cartilage‐centric [34]. Cartilage loss is a defining feature, but clinically meaningful OA involves coordinated changes across synovium, subchondral bone, meniscus, ligament, and periarticular tissues, with additional contributions from neuroimmune and vascular components that influence pain and inflammation [35]. Many genetically supported mechanisms are therefore unlikely to operate uniformly across the joint [14]. For variant interpretation, this means that a locus‐to‐gene assignment is incomplete without defining the relevant anatomical compartment in which the genetic effect is most likely to operate [36]. For biomarker development, it means that the most informative readout may reflect synovial inflammatory activity, bone remodeling, or compartment‐specific tissue stress rather than a generic “cartilage marker,” and that sampling feasibility varies substantially between joint compartments [37].

### 4.2. Single‐Cell Atlases Resolve the Cellular Sources of Genetic Signals

Bulk profiling averages across cell populations and can blur cell‐type–specific effects, which is particularly problematic when genetic regulation is state dependent [38]. Single‐cell transcriptomic and epigenomic atlases reduce this ambiguity by identifying which cell populations express candidate effector genes and which cellular states activate genetically supported programs [39]. In OA, this has shifted interpretation away from single marker genes toward cell‐state programs with clearer biological and biomarker relevance. Chondrocytes occupy multiple states linked to matrix synthesis, stress responses, hypertrophy‐like programs, and inflammatory signaling; synovial fibroblasts show distinct activation states associated with extracellular matrix remodeling and cytokine networks; and immune populations include macrophage and T cell subsets with variable inflammatory and antigen‐presentation features [40]. Placing genetically implicated genes within these state landscapes helps clarify whether a signal is more likely to reflect baseline susceptibility, active disease biology, or a response to tissue injury and remodeling—distinctions that influence whether a biomarker is suited for risk assessment, disease monitoring, or mechanistic stratification [41].

### 4.3. Spatial Profiling Anchors Mechanisms to Microanatomic Niches

Single‐cell data often lose information about where cells reside and which microenvironmental cues shape their behavior [42]. Spatial transcriptomics and imaging‐based molecular profiling recover this information by mapping programs to specific joint compartments and lesion niches [13]. This is particularly valuable in OA because disease processes are focal and patterned: cartilage lesions, osteophyte margins, tidemark changes, synovial lining alterations, and subchondral sclerosis are not distributed evenly [43]. Spatial localization can reconcile apparently inconsistent findings across cohorts by revealing that a program may be strong but restricted to a compartment that is under‐sampled or diluted in bulk tissue [36]. For translation, spatial information also clarifies which clinically measurable proxies are most biologically justified [44]. Programs localized to synovial lining regions are more likely to yield informative synovial fluid and inflammatory protein signatures, whereas remodeling programs concentrated in subchondral niches may align better with imaging phenotypes and bone‐associated molecular measures [45].

### 4.4. Selecting Clinically Measurable Surrogates for Tissue‐Anchored Programs

Context mapping is useful only if it informs what can be measured in practice. Joint tissues are rarely sampled in early disease, so translation typically relies on surrogates—synovial fluid analytes, circulating proteins or metabolites, extracellular vesicle cargo, and imaging‐derived features that reflect structural or compositional change [46]. The goal is not to maximize the number of features, but to choose a small set that consistently tracks the tissue‐anchored program of interest and remains stable across cohorts and platforms [47]. This prioritization favors biomarkers that balance biological specificity with practical measurability. This is also where genetics and joint atlases can improve multimodal modeling: when candidate features are tied to a plausible tissue and cellular mechanism, resulting signatures tend to be more interpretable and are less likely to hinge on cohort‐specific artifacts. In OA, where both biological heterogeneity and practical sampling constraints are substantial, aligning biomarkers to tissue‐ and state‐resolved mechanisms is often the difference between an elegant discovery result and a deployable clinical tool [48].

## 5. Designing Genetics‐Informed Biomarkers: From Mechanisms to Measurable Readouts

### 5.1. Polygenic Scores as Tools for Early Stratification

PRS offer a direct way to summarize inherited liability and can be useful even when they do not achieve diagnostic performance on their own [49]. In OA, PRS are best positioned as risk enrichment tools rather than diagnostic markers, identifying individuals with higher baseline risk who may benefit from earlier monitoring, prevention‐oriented counseling, or inclusion in studies where higher event rates improve power [50]. PRS can also support endotyping when combined with joint‐site information and intermediate phenotypes, although this requires careful phenotype definition and validation [51]. A key practical point is that PRS typically capture long‐term susceptibility, whereas near‐term progression and symptom trajectories are more strongly influenced by current tissue state, biomechanics, comorbidities, and local inflammation [52]. Accordingly, PRS are most informative when interpreted alongside molecular or imaging indicators of current disease activity rather than as stand‐alone predictors.

### 5.2. Compact Molecular Panels That Reflect Genetically Supported Programs

Genetics can improve biomarker design by narrowing attention to molecular features that plausibly sit close to causal mechanisms [53]. Rather than assembling large panels that are difficult to reproduce, a more deployable approach is to define compact panels that capture specific, tissue‐anchored programs supported by genetic and functional evidence [54]. Proteomic and metabolomic assays are attractive because they can be standardized and scaled, and extracellular vesicles provide an additional route to access tissue‐derived molecular cargo with minimally invasive sampling [48]. The optimal panel composition will therefore depend on the biological program being targeted rather than on maximizing marker number. The most plausible panel content will differ by mechanism: inflammation‐linked programs may be reflected by synovial fluid or circulating immune mediators; cartilage matrix turnover may be captured by specific degradation fragments or modification patterns; and subchondral remodeling may be reflected by bone‐associated proteins and metabolites [55]. Even when candidate molecules are measurable, deployment depends on whether they show stable performance across cohorts and whether they add value beyond readily available clinical variables [56].

### 5.3. Imaging–Omics Models that Connect Structure to Mechanism

Imaging captures the spatial and structural expressions of OA, whereas omics profiles can reflect underlying molecular activity [57]. Their integration therefore provides a natural route to mechanistically interpretable multimodal biomarkers [58]. Genetics can strengthen this integration by guiding attention toward imaging features that align with genetically supported biology and away from unstable correlates [51]. For example, imaging patterns suggestive of synovitis or effusion may pair naturally with inflammatory protein signatures, whereas subchondral bone changes may align with remodeling‐associated molecular measures [59]. Multimodal models and AI‐based approaches can be powerful in this setting, but they are vulnerable to domain shift across scanners, acquisition protocols, segmentation pipelines, and healthcare settings [60]. Mechanism alignment improves interpretability, but transportability still requires explicit cross‐site validation and careful control of technical variation.

### 5.4. Defining Intended Use and Evaluating Clinical Value

A common reason biomarkers fail to translate is that discovery is performed without a clear clinical decision point in mind [61]. Genetics‐informed biomarkers should be designed for a specific use: risk stratification before symptoms, prediction of structural progression, identification of mechanism‐linked endotypes, or selection and monitoring of patients in clinical trials [62]. The intended use determines acceptable assay complexity, sampling frequency, and the level of performance required for clinical relevance. Evaluation should extend beyond association and discrimination to include calibration and measures that reflect clinical impact, such as whether the biomarker improves decisions relative to standard clinical predictors and imaging [63]. In OA, near‐term utility is often strongest for tools that enable earlier monitoring in high‐liability individuals or enrich trials for mechanism‐relevant subgroups, because these applications have clearer action pathways than replacing imaging or clinical assessment outright [64].

## 6. Pitfalls and Failure Modes in Translation

### 6.1. Causal Uncertainty and Limited Joint‐Relevant Functional Evidence

Even when OA associations are robust, causal interpretation often remains uncertain [65]. Linkage disequilibrium can leave multiple variants plausible, and effector gene assignment is frequently ambiguous for regulatory signals that act over distance or only in specific cellular states [21]. In OA studies, this uncertainty is often compounded by reliance on functional datasets derived from nonjoint tissues or unrelated disease contexts. A common weakness in OA translation is over‐reliance on functional resources that do not match the joint environment, or on bulk measurements that average heterogeneous cell populations [66, 67]. These mismatches can yield confident but fragile conclusions, especially for effects that are restricted to particular compartments or disease stages. As a result, biomarker candidates may be built on variant‐to‐gene links that do not hold once the correct tissue context is examined.

### 6.2. Tissue Access, Disease Stage, and Surrogate Sampling Constraints

Access to joint tissues in early OA remains extremely limited, and most high‐resolution molecular studies rely on specimens obtained during joint replacement surgery, which predominantly represent advanced disease [68]. This introduces an inherent stage bias, as molecular features identified in late‐stage tissue may reflect consequences of disease progression rather than early pathogenic drivers [69]. In contrast, more accessible biospecimens such as peripheral blood often capture systemic influences and comorbid conditions rather than joint‐specific biology, whereas synovial fluid—although biologically informative—is not routinely obtained outside specialized clinical settings [70]. Together, these practical constraints complicate the selection of biomarkers that are both biologically relevant and feasible for routine clinical measurement. Consequently, some candidate markers may show strong biological rationale yet prove difficult to implement consistently across populations, joint sites, or disease stages [71].

### 6.3. Technical Confounding and Reproducibility Challenges in Multiomics and Imaging

Multiomics analyses are particularly vulnerable to technical variability arising from batch effects, differences in analytical platforms, and preanalytical factors such as sample processing and storage conditions [72]. Similarly, imaging‐based biomarkers may be influenced by heterogeneity in scanner hardware, acquisition protocols, image segmentation approaches, and feature extraction pipelines [73]. When these sources of variation coincide with study design factors such as recruitment site, disease severity, or case‐control structure, they may introduce spurious associations and reduce the stability of findings in independent cohorts [74]. These risks are further amplified in OA research, where tissue‐based datasets are often limited in size but high in dimensionality. Addressing these challenges requires rigorous quality control procedures, strict separation between training and validation analyses, and external validation strategies that account for the heterogeneity encountered in routine clinical environments [75].

### 6.4. Generalizability and the Clinical‐Utility Gap

Strong performance in a discovery cohort does not necessarily translate into clinical usefulness if a biomarker cannot maintain performance across diverse populations or healthcare contexts [76]. For example, PRS frequently show reduced predictive performance when applied to populations with different ancestral backgrounds, whereas molecular and imaging signatures may be influenced by differences in demographic structure, comorbidity burden, or patterns of clinical care [27]. Moreover, statistical significance alone does not ensure clinical relevance [77]. In OA, biomarkers are most likely to have practical value when they provide information beyond established clinical and imaging predictors and when their results can support actionable decisions, such as identifying individuals who may benefit from closer monitoring, recognizing patients at risk of accelerated progression, or improving selection of participants for mechanism‐driven clinical trials [78]. Without a clear link to clinical decision‐making, even well‐performing biomarkers may ultimately have limited impact on patient management [79].

## 7. Implementation and Governance Considerations

Clinical adoption of genetics‐informed biomarkers in OA depends less on additional discovery and more on rigorous validation, assay standardization, and integration into real‐world clinical workflows [80]. Validation should be aligned to a clearly defined use‐case—screening for long‐term susceptibility, forecasting near‐term progression, classifying endotypes, or predicting response—and should use harmonized phenotypes and follow‐up windows across cohorts [81]. External replication across sites and populations is essential, and once thresholds are proposed, prospective evaluation becomes important because performance estimates from retrospective studies often degrade in routine care [82]. Reporting should emphasize calibration and clinical impact alongside discrimination, since decision‐making requires reliable risk estimates and evidence that the biomarker can meaningfully influence clinical management rather than simply correlate with disease status [83].

Implementation also requires practical decisions about what is feasible to measure and how results will be used [84]. PRS‐based stratification is most realistic for earlier monitoring, prevention‐oriented counseling, or trial enrichment rather than immediate treatment selection [85]. Molecular panels must match sample accessibility and intended timing, with clear justification for whether they represent a relatively stable endotype or a dynamic activity state [86]. Imaging‐derived measures depend on standardized acquisition and robust feature extraction; without harmonization, cross‐site variability can overwhelm biological signal. Across all modalities, outputs should be communicated in forms that map to clinical actions, rather than as isolated continuous scores without an associated clinical interpretation framework.

Finally, responsible implementation also requires careful attention to governance frameworks [87]. The use of genetic and multiomics data raises important considerations related to data privacy, informed consent, secure data sharing, and traceable model governance, particularly when these datasets are integrated with longitudinal clinical information and imaging resources. For multimodal prediction systems, issues of fairness and accountability should be addressed through validation across diverse patient populations, transparent reporting of model uncertainty and limitations, and predefined strategies for performance monitoring and model updating. Ultimately, these governance and implementation factors play a decisive role in determining whether genetics‐informed OA biomarkers can move beyond research settings and become clinically credible, interpretable, and practically usable tools.

## Author Contributions

T.M., L.M., and A.L. conceived and designed the study. T.M., L.M., X.Z., J.H., F.L., W.W., S.L., and A.L. drafted the manuscript. T.M. and L.M. performed literature retrieval and data collection. A.L. supervised the project, critically revised the manuscript, and approved the final version. T.M., and L.M. have contributed equally to this work and share first authorship.

## Funding

This study was supported by the Innovation Team Cultivation Program of the First Teaching Hospital of Tianjin University of Traditional Chinese Medicine, 4042502041.

## Disclosure

All authors read and approved the final manuscript.

## Conflicts of Interest

The authors declare no conflicts of interest.

## Data Availability

Data sharing not applicable to this article as no datasets were generated or analyzed during the current study.
